# Social robot PIO intervention for improving cognitive function and depression in older adults with mild to moderate dementia in day care centers: A randomized controlled trial

**DOI:** 10.1371/journal.pone.0321745

**Published:** 2025-04-22

**Authors:** Jun-Seo Lim, Hye-Kyung Oh

**Affiliations:** 1 Department of Nursing, Bucheon University, Bucheon, South Korea; 2 College of Nursing, Daegu University, Daegu, South Korea; University of Auckland, NEW ZEALAND

## Abstract

The increases in the older population, the prevalence of dementia, and the resulting social costs are burdensome to individuals, families, and the nation. This study examines whether the social robot PIO program intervention is effective for cognitive function and depression for older adults with mild to moderate dementia using two daycare centers in Daegu, Korea. Older adults with mild to moderate dementia and using a daycare center were included in the experimental (n = 33) and control (n = 33) groups. The experimental group participated in the social robot PIO program twice a week, 12 sessions, 50 minutes day for 6 weeks, and the control group received the usual care. From October 2022 through December 2022, a total 66 participants were included. Results showed that the cognitive function of the experimental group increased by 3.9±3.66, from 18.1±4.54 before intervention to 21.9±5.17 after intervention; the control group increased by 0.1±4.13, from 18.2±4.91 before intervention to 18.2± 4.77 after intervention. The difference between the two groups was statistically significant (*t* = 3.94, *p*<.001). Depression decreased -0.7±3.48 in the experimental group, from 5.9±4.74 before intervention to 5.2±4.65 after intervention, and decreased by -0.2±3.42, from 6.5±4.69 before intervention to 6.4±4.08 after intervention, in the control group, but the difference between the two groups was not statistically significant (*z* = -0.59, *p* =.557). It was confirmed that the social robot PIO program is effective in improving cognitive function in older people with mild to moderate dementia who use daycare centers. Therefore, it is recommended to periodically implement this program for the older adults who use daycare centers to improve cognitive function. The experimental group had lower depression than did those in the control group, but the effect is not statistically significant, so additional research is required.

**Trial Registration:** CRIS (KCT0007936)

## Introduction

It has been estimated that 55.2 million people worldwide suffered from dementia in 2019, about 78 million people will do so in 2030, and about 139 million people in 2050 [1]. In 2020, the estimated prevalence of dementia among those aged 65 years or older in the Republic of Korea was 10.3% (approximately 840,000 people), and the prevalence in 2050 is estimated to be 15.9% (3.02 million people) [2]. The global cost of dementia in 2019 was estimated to be USD 1.3 trillion and is projected to rise to USD 1.7 trillion by 2030 [1]. Dementia care costs in Korea totaled USD 12.4 billion in 2019, accounting for about 0.9% of GDP, and are expected to increase to USD 77.8 billion in 2050, accounting for about 3.8% of GDP [3]. As such, the increases in the older population, the prevalence of dementia, and the resulting social costs are burdensome to individuals, families, and the nation.

Intervention approaches for older adults with dementia can be divided into pharmaceutical interventions (PIs) and non-pharmacological interventions (NPIs). PIs include cholinesterase inhibitors or N-methyl-D-aspartate receptor antagonists to improve cognitive function, and neuroleptics, anti-anxiety drugs, and antidepressants to improve behavioral psychological symptoms [4–6]. Although PIs can alleviate symptoms, they do not provide a fundamental treatment for dementia and can cause side effects such as delirium and agitation [5,6]. NPIs are not disease-specific and are less likely to cause side effects [7]. NPIs can be classified into standard therapies, alternative therapies, and brief psychotherapy depending on treatment goal. Several NPIs being used in combinations [8,9]. Since pharmacological treatment for dementia can produce various side effects, non-pharmacological treatment is preferentially proposed [8,10].

Recently, a social robot was used as an NPI and was reported to have positive effects on cognitive function, depression and loneliness, and communication in older people with dementia [10,11]. However, when looking at previous studies of social robot interventions using pet-type social robots targeting this population, the effects were inconsistent [12–15]. Pet-type social robots interventions aim to replace a live animal with a social robot in animal assisted therapy (AAT). AAT and pet robot assisted therapy seem to reduce agitation and have a positive effect on social interaction and mood disorders, but no effect was observed on cognitive function [16–18].

Computer-based rehabilitation (CBR) is being used to improve cognitive function and prevent cognitive decline in older adults with mild cognitive impairment (MCI) and dementia, utilizing computer programs such as RehaCom, ComCog, and CoTras [19]. CBR intervention not only helps improve cognitive function, memory, and attention, but also has a positive effect on psychosocial functions such as depression and loneliness in older adults with MCI [20]. Sil-bot is a humanoid-type social robot equipped with a CBR program. Studies using Sil-bot in interventions with MCI patients reported reduced cortical thinning and depression and improved cognitive function [21,22]. Therefore, to compensate for the limitations of pet-type social robots’ influence on cognitive function variables, the combination of CBR and active cognitive intervention programs is required. However, it is difficult to find studies combining pet-type social robots and CBR. The social robot PIO used in this study is a parrot-type social robot for increasing positive social interaction and improving mood disorders; it is also equipped with a CBR program to improve cognitive function in older people with dementia.

This study aimed to confirm the positive effects of intervention using the social robot PIO, equipped with a parrot-type CBR program, on cognitive function and depression in older adults with mild to moderate dementia.

## Materials and methods

### Study design

This study uses a randomized controlled trial design to determine whether an intervention using the social robot PIO is effective for improving cognitive function and depression in older people with mild to moderate dementia.

### Participants

**Fig 1:**
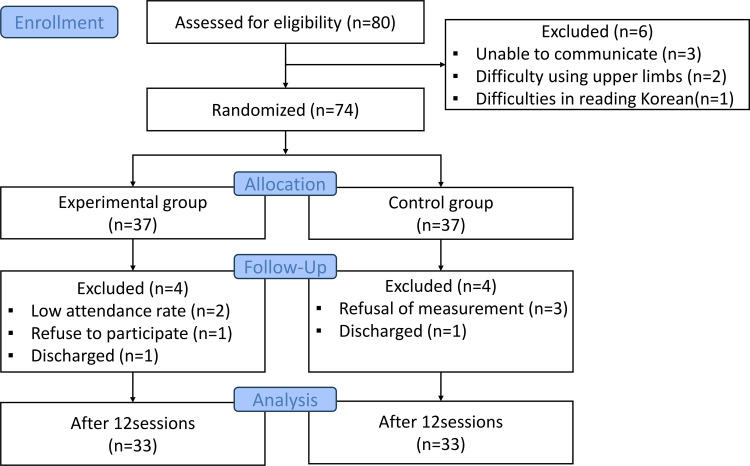
CONSORT flow diagram of participants through the trial.

Participants were recruited in two daycare centers in Daegu, South Korea. Inclusion criteria were: aged 65 years or older, probable cognitive impairment as rated by the Cognitive Impairment Screening Test, which is used as a screening tool for dementia in Korea, able to read and communicate in Korean, without impairment to upper limb and body mobility, vision, and hearing. Before intervention, it was not necessary to assess the capacity to consent, because, participants were cognitively competent to express their willingness, according to a screening tool for dementia used in the inclusion criteria, and the ethics committee approved to follow this procedure. Their Exclusion criteria were psychiatric history such as schizophrenia and bipolar disorder, unable to communicate due to severe cognitive impairment, impaired use of their upper limbs, vision, or hearing (Fig 1). The study was registered with the Clinical Research Information Service (CRIS KCT0007936) for clinical trials in Republic of Korea. The authors confirm that all ongoing and related trials for this drug/intervention are registered. The research was conducted from October 17 to December 15, 2022.

### Interventions

**Fig 2:**
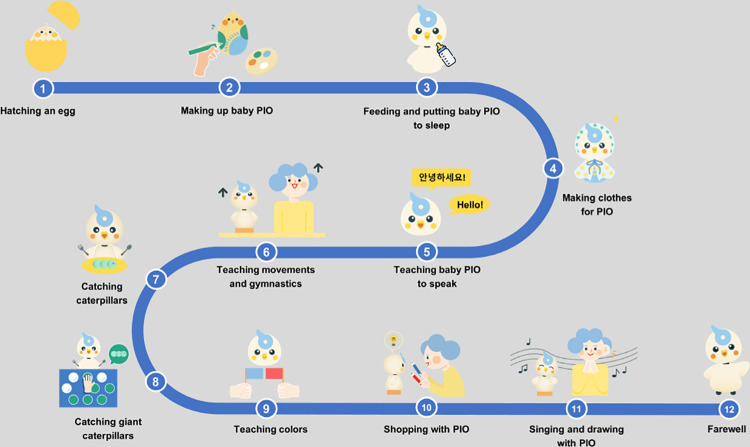
12 session programs of social robot PIO.

The social robot PIO program consists of 12 (50-minute per each) sessions, held twice a week for 6 weeks based on the storytelling of “hatch from an egg and grow into an adult social robot.” Each session of the PIO intervention was conducted with groups of less than 10 participants at a time. The social robot PIO program had a 1:1 ratio (participant: robot). The program was conducted with the principal researcher and two assistant researchers. All sessions consisted of three stages: introduction, intervention, and conclusion. The introduction and conclusion consist of meeting and saying farewell to the PIO robot. In the intervention stage, therapeutic factors such as music therapy, art therapy, occupational therapy, gymnastics, sensory stimulation, and recall therapy were configured according to the theme of each session (Table 1, Fig 2). The control group did not perform any new interventions related to cognition or depression during the intervention period and maintained their usual cares. The content of the PIO program is provided in multimedia appendixes.

**Table 1:** Contents of PIO Program.

Session	Topics	Contents
1	**Hatching an egg**	Introduction
		Stretching exercise
		Shaking an egg to hatch
2	**Making up baby PIO**	Stretching exercise
		Makeover a baby PIO
		Decorating nest for baby PIO
3	**Feeding and putting a baby PIO to sleep**	Stretching exercise
		Feeding milk with formula
		Rock a baby PIO to sleep
4	**Making clothes for PIO**	Stretching exercise
		Making clothes
5	**Teaching baby PIO to speak**	Stretching exercises
		Finding proper words for specific situations
6	**Teaching movements and gymnastics**	Stretching exercises
		Teaching movements
		Gymnastics with PIO
7	**Catching caterpillars**	Gymnastics with PIO
		Catching caterpillars
8	**Catching giant caterpillars**	Gymnastics with PIO
		Catching a moving giant caterpillar
9	**Teaching colors**	Gymnastics with PIO
		Teaching color words
		Coloring and catching caterpillars
10	**Shopping with PIO**	Gymnastics with PIO
		Shopping at the supermarket
		Matching scenes sequences
		Remember locations
11	**Singing and drawing with PIO**	Gymnastics with PIO
		Clapping with PIO
		Drawing portrait
12	**Farewell**	Gymnastics with PIO
		Recalling the entire program
		Sharing your feelings

### Blinding

This study was a double-blind clinical trial. The participants were unaware of being involved in either group. During measurement, the data were assessed by research assistants who were unaware of participants’ assignment into the control or intervention groups. Participants were clustered by age and divided into two groups using an online research randomizer (http://www.randomizer.org) by researchers independent of the study.

### Measurements

The six factors concerning participants’ personal characteristics were constructed by researchers based on previous studies, and focused on gender, age, marital status, education, religion, and number of chronic diseases. Measurements of each instrument were performed before the start of the first session and after the end of the last session.

### Cognitive function

Cognitive function was measured using the Korean Mini-Mental State Examination, 2nd Edition (K-MMSE~2): SV (Standard Version) by Kang et al. [23], which translated the 2010 revised Mini-Mental State Examination (MMSE) developed by Folstein et al. [24]. The maximum score for MMSE is 30, with lower scores indicating greater impairment. It consists of registration, orientation to time and place, recall, attention and calculation, language, and drawing. Regarding this scale’s reliability at the time of development, Cronbach’s α for the original scale was.69. In this study, Cronbach’s α was.75.

### Depression

Depression was measured using the 15- item Geriatric Depression Scale Short Form: Korean Version (GDSSF-K), developed by Yesavage and Sheikh [25] and translated and modified by Kee [26]. This scale is rated on a dichotomous scale (0 = ‟yes”, 1 = ‟no”). Higher scores indicate more depression. Cronbach’s α for the original scale was.88. In this study, Cronbach’s α was.90.

### Sample size

The sample size was determined using the G-Power 3.1.9.2 program, with a significance level of 0.05, a power of 0.8, two outcome variables (cognitive function and depression), and an effect size of 0.7, and the minimum sample size was calculated to be 68 people. Considering the dropout rate, a total of 80 participants were recruited. A researcher who was not involved in this study stratified the subjects according to gender, age, and education level, assigned numbers in order, and then randomly assigned them into the experimental and control groups using an online service provided by Social Psychology Network’s research randomizer. Among the experimental (n = 37) and control (n = 37) groups, four participants in each group were dropped due to not participating in the program for more than three sessions, refusal to participate, and leaving the daycare center. Finally, 33 participants in the experimental and 33 in the control group completed all 12 sessions.

### Data collection

Data were collected from October 17, 2022 to December 15, 2022. Recruitment notices were posted on bulletin boards at two daycare centers in Daegu, Korea. Older adults who wanted to participate and their caregivers were contacted to collect basic information, and the assistant researcher explained the purpose of the study to the subjects and their guardians and obtained informed consent. Measurements started at baseline and ended after 6 weeks.

### Social robot PIO

The social robot PIO used in this study is version 3.4, developed by Whydots Co., Ltd. in Korea for older adults with cognitive impairment, and its appearance is in the form of a parrot.

**Fig 3:**
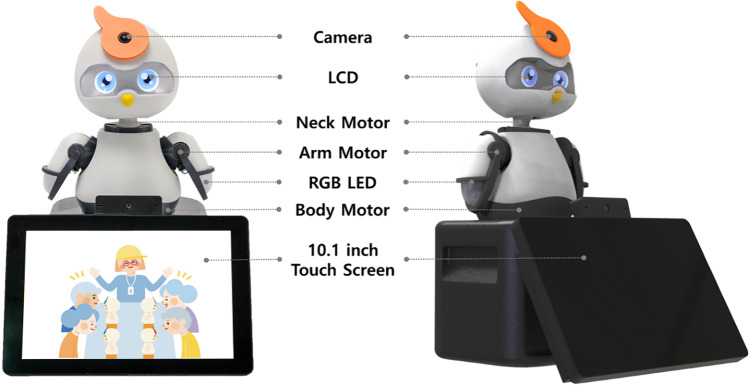
Social robot PIO (version 3.5).

Unlike earlier versions, version 3.4 has a tablet PC integrated with the body. The size of the social robot PIO is 265mm×260mm×370mm (Width×length×height) and weighs about 2.2 kg. It mimics various emotions using seven facial expressions and six degrees of freedom (Fig 3).

### Statistical analysis

The data were analyzed using STATA 17. Descriptive statistics were used for demographic characteristics. The t test was used to compare the means of the continuous variables of the two groups, and the chi-square and Fisher tests were used to test the homogeneity of proportions between the two subgroups. The test for the main variables to confirm the effect of the program was analyzed using independent t-test (two-tailed test) in case of normal distribution and Mann-Whitney U test in case of non-normal distribution. The significance level was set at *p* <.05. Data analysis was conducted by a statistical analyst for objective results.

### Ethics

This study was undertaken after obtaining approval from the Institutional Review Board (No. 1040621–202209-HR-079) of D University in Republic of Korea. We explained the study’s purpose, methods, and procedure; the confidentiality and anonymity of the data; and the fact that the subjects could withdraw at any time for any reasons. Subsequently, written consent to participate was obtained.

## Results

### Participant characteristics

From October 2022 through December 2022, a total 66 older adults with mild to moderate dementia were included. Participants’ demographic characteristics are summarized in Table 2. There were 27 females (81.8%) and 6 males (18.2%) in the experimental group, and 24 females (72.7%) and 9 males (27.3%) in the control group. Participants’ average age was 82.9 ± 5.45(Mean ± SD) years in the experimental group and 84.4 ± 4.94 years in the control group. In the experimental group, 13 (39.4%) were 85–89 years old, and 12 (36.4%) were 85–89 years old in the control group. Regarding marital status, 24 (72.7%) of the experimental group and 25 (75.8%) of the control group were widowed. As for education level, “no education” was the most common answer in experimental group (n = 12, 36.4%), and “elementary school” was the most common response in the control group (n = 16, 48.5%) had completed in control group. There were 17 participants (51.5%) in the experimental group and 16 (48.5%) in the control group with two or more chronic diseases.

**Table 2:** Demographic characteristics of participants.

Characteristics	Variables	EXP (n=33), n (%)	CON (n=33), n (%)	*p* value
**Gender**	Male	6 (18.2)	9 (27.3)	.38
	Female	27 (81.8)	24 (72.7)	
**Age**	70~74	2 (6.1)	2 (6.1)	.80^†^
	75~79	6 (18.1)	4 (12.1)	
	80~84	10 (30.3)	10 (30.3)	
	85~89	13 (39.4)	12 (36.4)	
	90~	2 (6.1)	5 (15.1)	
	Mean±SD	82.9±5.45	84.4±4.94	.25
**Marital status**	Single	0 (0.0)	0 (0.0)	1.00^†^
	Married	8 (24.2)	8 (24.2)	
	Separated/Divorce	1 (3.1)	0 (0.0)	
	Widowed	24 (72.7)	25 (75.8)	
**Education**	No Education	12 (36.4)	4 (12.1)	.12^†^
	Elementary	9 (27.3)	16 (48.5)	
	Middle	5 (15.2)	7 (21.2)	
	High	4 (12.1)	5 (15.2)	
	College	3 (9.0)	1 (3.0)	
**Religion**	Buddhist	12 (36.4)	11 (33.3)	.78^†^
	Christian	8 (24.2)	7 (21.2)	
	Catholic	1 (3.0)	3 (9.1)	
	No religion	12 (36.4)	12 (36.4)	
**Number of Chronic Diseases**	1	16 (48.5)	17 (51.5)	.81
	≥2	17 (51.5)	16 (48.5)	

EXP: experimental group; CON: control group; SD: standard deviation;

† Fisher’s exact test (two-tailed test)

### Effects of PIO interventions on cognitive function

Analysis of the difference in total scores on cognitive function before and after the intervention between the experimental and the control groups indicated that the experimental group increased by 3.9±3.66, from 18.1±4.54 before the intervention to 21.9±5.17 after the intervention, and the control group increased by 0.1± 4.13, from 18.2±4.91 before the intervention to 18.2±4.77 after the intervention. The change in total scores from before and after the intervention for the experimental and control groups was statistically significant (*t* = 3.94, *p* <.001). In the subdomains, orientation to time (*t* = 4.15, *p* <.001) and language (*z* =2.17, *p* =.034) were s*t*atistically significant, but registration (z = 0.86, *p* =.392), orientation to place (*t* = 1.80, *p* =.076), recall (*t* = 0.65, *p* =.515), a*tt*ention and calculation (*t* =1.53, *p* =.130), and drawing (*t* = 0.00, *p* =1.000) were no*t* s*t*atistically significant (Table 3).

**Table 3:** Comparison of cognitive function between the experimental and control groups.

Variables	Groups	Pre-test		Post-test		Post-pre test		*p* value
		Mean± SD	Median (IQR)	Mean ± SD	Median (IQR)	Mean ± SD	Median (IQR)	
**Total**	EXP (n=33)	18.1±4.54	18 (7)	21.9±5.17	22 (8)	3.9±3.66	3 (4)	<.001
	CON (n=33)	18.2±4.91	17 (8)	18.2±4.77	19 (6)	0.1±4.13	0 (5)	
Registration	EXP (n=33)	2.6±0.94	3 (0)	2.8±0.46	3 (0)	0.2±0.90	0 (0)	.39
	CON (n=33)	2.8±0.48	3 (0)	2.8±0.61	3 (0)	0.0±0.73	0 (0)	
Orientation to time	EXP (n=33)	2.5±1.68	2 (3)	3.4±1.56	4 (3)	0.9±1.19	1 (1)	<.001
	CON (n=33)	2.5±1.56	3 (3)	2.2±1.46	2 (2)	-0.4±1.30	0 (1)	
Orientation to place	EXP (n=33)	3.8±0.99	4 (1)	4.2±0.79	4 (1)	0.5±1.09	0 (1)	.08
	CON (n=33)	3.8±1.13	4 (2)	3.8±1.03	4 (2)	-0.1±1.22	0 (2)	
Recall	EXP (n=33)	0.5±0.94	0 (1)	1.1±1.14	1 (2)	0.5±0.97	0 (1)	.52
	CON (n=33)	0.8±0.99	0 (2)	1.1±1.19	1 (2)	0.3±1.27	0 (1)	
Attention and Calculation	EXP (n=33)	2.0±1.51	2 (2)	2.7±1.86	3 (3)	0.7±1.68	1 (2)	.13
	CON (n=33)	1.7±1.69	1 (3)	1.8±1.46	1 (2)	0.2±1.35	0 (2)	
Language	EXP (n=33)	6.1±1.47	7 (2)	7.1±1.34	8 (1)	1.0±1.59	1 (2)	.03
	CON (n=33)	5.9±1.59	6 (2)	6.0±1.63	7 (2)	0.1±2.01	0 (2)	
Drawing	EXP (n=33)	0.6±0.50	1 (1)	0.6±0.49	1 (1)	0.0±0.64	0 (0)	1.00
	CON (n=33)	0.6±0.50	1 (1)	0.6±0.49	1 (1)	0.0±0.53	0 (0)	

EXP: experimental group; CON: control group; SD: standard deviation; IQR: Inter Quartile Range

### Effects of PIO interventions on depression

Analysis of the difference in scores before and after the intervention in the experimental group and the control group indicated that depression in the experimental group decreased by -0.7±3.48, from 5.9±4.74 before the intervention to 5.2±4.68 after the intervention, and the median and interquartile range (IQR) decreased by 0 (5), from 4 (8) before the intervention to 3 (6) after the intervention. The control group decreased -0.2±3.42, from 6.5±4.69 before intervention to 6.4±4.08 after intervention, and the median and IQR were 5 (8) before intervention to 6 (6) after intervention; the difference value before and after intervention was 0 (4). The difference between the two groups before and after intervention was not statistically significant (z = -0.59, *P* =.557) (Table 4).

**Table 4:** Comparison of depression between the experimental and control groups.

Groups	pre		post		post-pre		*p* value
	M±SD	Median (IQR)	M±SD	Median (IQR)	M±SD	Median (IQR)	
EXP (n=33)	5.9±4.74	4(8)	5.2±4.68	3(6)	-0.7±3.48	0(5)	0.56
CON (n=33)	6.5±4.69	5(8)	6.4±4.08	6(6)	-0.2±3.42	0(4)	

EXP: experimental group; CON: control group; SD: standard deviation

## Discussion

Our study revealed that CBR based robot PIO intervention contributed to a significant increase in participants’ cognitive function among older adults with mild to moderate dementia. Previous studies had varying results for cognitive function depending on what kind of social robot was applied. The humanoid-type social robot based on CBR can improve cognitive function in older adults with MCI [21]. In contrast, pet-type social robots had no statistically significant changes in cognitive function after intervention [16,17,27]. It is interpreted that pet-type social robots are primarily designed with a focus on psychological domains, such as agitation, anxiety, and stress, rather than focusing on cognitive function. Among humanoid type robots, an intervention with the NAO robot reported no statistically significant cognitive function changes [16]. Because, NAO also does not developped with a focus on CBR for improving cognitive function [28].

Traditional pen-and-paper cognitive rehabilitation conducted by a neuropsychologist is very costly [29]. In particular, timely treatment is crucial to achieve better outcomes and fewer complications, it is important to improve accessibility to services and treatments [30]. Robots designed to assist older adults may be important due to the rapid increase in the aging population and the exorbitant healthcare costs associated with caring for older individuals with cognitive decline [21]. CBR has advantages such as standardization of the administration and stimulus, automated real-time comparison with an individual’s prior performance, and efficiencies of staffing and cost [31]. Thus, CBR is an approach that utilizes computer technology to enhance the effectiveness of rehabilitation therapy and provide personalized care, offering the benefits of non-pharmacological intervention. Nevertheless, CBR-based cognitive programs still face the challenge of being accepted by older adults who have a difficult relationship with technology [32]. To solve the unfamiliarity issue, a CBR-based social robot called PIO was programmed based on storytelling for growth from egg to an adult parrot robot; each session contained therapeutic factors such as art, language, occupational therapy, etc., to increase immersion and bonding between older adults and PIO. Thus, the CBR-based social robots were programmed to talk, move, and express emotion, and could constitute a new alternative intervention to decrease dementia symptoms.

We reported that participants in the experimental group had lower levels of depression than did those in the control group, but the difference was not statistically significant. In a previous study, a humanoid-type social robot equipped with CBR had significantly greater positive effects in depression post-intervention [21]. Although these results cannot be easily interpreted because a different type of CBR-based social robot was applied, older adults with dementia may have decreased depression due to the possibility that the robots promoted interaction among participants while they shared a humanoid social robot during the intervention in Park and Jung [21]’s study. In the present study, it is interpreted that the non-significant effect on depression was due to the PIO robots and participants were matched 1:1, without interaction within the group. Among social robots, a seal-type PARO reacts to external stimuli with sensors and has tactile features (soft, fluffy surface) that encourage users to engage in physical interaction with robot [33]. This interaction is analyzed to have resulted in a significant reduction in participants’ depression. However, PIO was operated based on a structured program and has no sensors to react to external stimuli. Furthermore, active interaction with the soft, fluffy surface of PARO can reduce user’s depression level [34], PIO has not these characteristics. For PIO to have a significant impact on participants’ depression level, it needs to be equipped with tactile sensors that respond to external stimuli immediately and improved tactile aspects.

To the best of our knowledge, this study is the first to compare the effects of a parrot-type CBR-linked social robot in terms of cognitive function and depression in older adults with mild to moderate dementia. Additionally, our study underpins the potential use of digital devices in NPI strategies. Through CBR-linked robot interventions, it allows group intervention without a neuropsychologist and allows individual access to older adults with dementia. Therefore, CBR-linked robotic intervention is recommended as a novel, integrated approach to attenuate dementia in a super-aged society.

This study had some limitations. First, the sample size was small; therefore, further studies should include larger samples. Second, follow-up sessions should be considered to confirm the persistent effects of program. Lastly, we did not consider to understand participants’ perceived usability of social robot intervention. For individual goal setting, it is necessary to assess individuals’ acceptability and usability of the technology before the intervention. Additional study should include a usability assessment to evaluate impacts of intervention. Despite these limitations, to our knowledge, our study is the first to compare the effects of a parrot-type CBR-based social robot on cognitive function and depression of older adults with mild to moderate dementia. The social robot PIO intervention showed positive effects on cognitive function. In the future, it can be used to confirm the degree of improvement in patients’ cognitive function in daycare centers, welfare centers, and hospitals. Also, it is essential to evaluate various aspects, including intrinsic capacity in order to reflect the diverse needs of the older population [35]. Furthermore, it allows therapeutic intervention with individuals and groups with dementia, without a neuropsychologist. Therefore, we strongly recommend that CBR-based robot interventions could be a new integrated approach to solve the pending issue of dementia care during super-aged society.

## Conclusions

First, statistically significant improvement in cognitive function of older adults with mild to moderate dementia was noted. Unlikely, traditional cognitive rehabilitation, social robot PIO features storytelling to facilitate growth, it is designed that participants can immerse themselves and feel a sense of accomplishment at each stage. It can contribute to enhancing the motivation for continuous learning.

Second, there was no statistically significant effect on depression. Further research is needed to include group interventions to increase the interaction among participants, while considering the participants’ baseline level of dementia and depression.

## Supporting information

S1 File Questionnaire (Korean). (DOCX)

S2 File Questionnaire (English). (DOCX)

S3 File CONSORT checklist. (DOCX)

S4 File Trial study protocol. (PDF)

## Abbreviations

AAT: animal-assisted therapy
CBR: computer-based rehabilitation
GDSSF-K: geriatric depression scale short form Korean version
NPIs: non-pharmacological interventions
MCI: mild cognitive impairment
PIs: pharmaceutical interventions.
