# Supplementary material for: Social robot PIO intervention for improving cognitive function and depression in older adults with mild to moderate dementia in day care centers: A randomized controlled trial
Source: PLoS One. 2025 Apr 22;20(4):e0321745. doi: 10.1371/journal.pone.0321745 (PMC12013943; doi:10.1371/journal.pone.0321745)
Supplement: S1 File — (DOCX) [file pone.0321745.s001.docx]

설문조사

| **대상자 이니셜** | **대상자 식별번호** |
| --- | --- |
|  | - |

| **1회차 설문일자** |  | **202 년** | **월** | **일** |
| --- | --- | --- | --- | --- |
| **2회차 설문일자** |  | **202 년** | **월** | **일** |

| **연구책임자** |  | **조사원** |  |
| --- | --- | --- | --- |
| **서명** |  | **서명** |  |

**인구사회학적 특성**

| 1. 귀하는 | 몇 년 몇 월생입니까? | ( )년 ( | )월 |
| --- | --- | --- | --- |
| 2. 귀하의  ① 남성 | 성별을 무엇입니까?  ② 여성 |  |  |
| 3. 귀하의  ① 미혼 | 결혼 상태에 대해 표시하세요.  ② 배우자 있음 ③ 별거/이혼 | | ➃ 사별 |

1. 귀하의 자녀는 어떻게 되십니까?

① 있음(아들: 명, 딸: 명) ② 없음

1. 귀하의 최종학력은 어떻게 되시나요?

① 무학 ② 초등학교 졸업 ③ 중학교 졸업 ➃ 고등학교 졸업 ⑤ 대학교 졸업

1. 귀하의 종교는 무엇입니까?

① 있음(종교: ) ② 없음

1. 귀하가 현재 가지고 있는 질환이 있으신가요?

① 있음(질환명: ) ② 없음

1. 귀하는 신체적 장애가 있나요?

① 장애있음 ② 장애없음

1. 귀하는 수급 대상자입니까?

① 비해당 ② 수급자 ③ 차상위 계층

**한국어판 간이정신상태검사 2판(K-MMSE-2)**

**
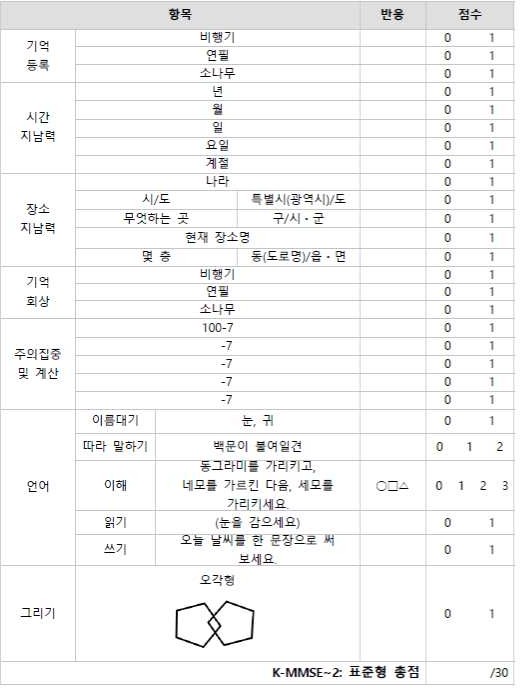
**


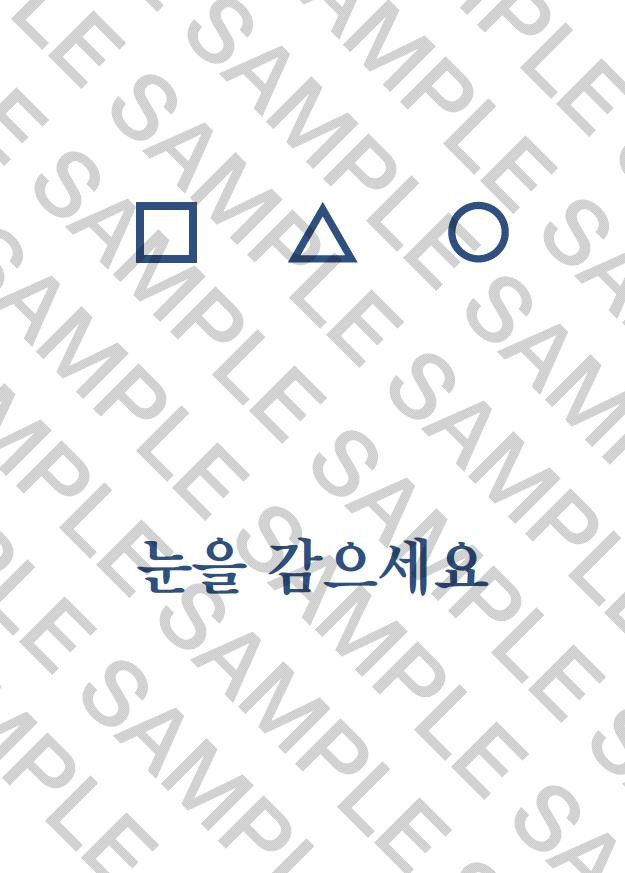


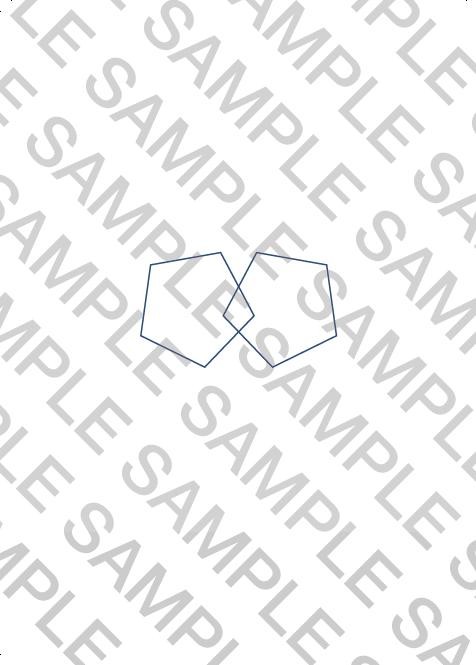


**한국형 노인우울척도 단축형(K-GDS)**

| **지난 1주일 동안의 느낌을 ⑨각하시면서 대답해주시기 바랍니다.** | **예** | **아니오** |
| --- | --- | --- |
| **1. 당신은 평소 자신의 ⑨활에 만족합니까?** | **①** | **②** |
| **2. 당신은 활동과 흥미가 많이 저하되었습니까?*** | **①** | **②** |
| **3. 당신은 앞날에 대해서 희망적입니까?** | **①** | **②** |
| **4. 당신은 대부분의 시간을 맑은 정신으로 지냅니까?** | **①** | **②** |
| **5. 당신은 대부분의 시간이 행복하다고 느끼십니까?** | **①** | **②** |
| **6. 당신은 지금 살아있다는 것이 아름답다고 ⑨각합니까?** | **①** | **②** |
| **7. 당신은 가끔 낙담하고 우울하다고 느낍니까?*** | **①** | **②** |
| **8. 당신은 지금 자신의 인⑨이 매우 가치가 없다고 느끼십니까?*** | **①** | **②** |
| **9. 당신은 인⑨이 매우 흥미롭다고 느끼십니까?** | **①** | **②** |
| **10. 당신은 활력이 충만하다고 느끼십니까?** | **①** | **②** |
| **11. 당신은 자주 사소한 일에 마음의 동요를 느끼십니까?*** | **①** | **②** |
| **12. 당신은 자주 울고 싶다고 느낍니까?*** | **①** | **②** |
| **13. 당신은 아침에 일어나는 것이 즐겁습니까?** | **①** | **②** |
| **14. 당신은 결정을 내리는 것이 수월합니까?** | **①** | **②** |
| **15. 당신의 마음은 이전처럼 편안합니까?** | **①** | **②** |
