# Supplementary material for: Social robot PIO intervention for improving cognitive function and depression in older adults with mild to moderate dementia in day care centers: A randomized controlled trial
Source: PLoS One. 2025 Apr 22;20(4):e0321745. doi: 10.1371/journal.pone.0321745 (PMC12013943; doi:10.1371/journal.pone.0321745)
Supplement: S2 File — (DOCX) [file pone.0321745.s002.docx]

# Survey

| Participant Initials | Participant Identification Number |
| --- | --- |
|  | - |

| First Survey Date |  | **202 /** | **/** |  |
| --- | --- | --- | --- | --- |
| Second Survey Date |  | **202 /** | **/** |  |

| Principal Investigator |  | Surveyor |  |
| --- | --- | --- | --- |
| Signatures |  | Signatures |  |

## Demographic Characteristics

1. What is your birth year and month?

( ) Year ( ) Month

2. What is your gender?

① Male ② Female

3. What is your marital status?

① Single ② Married ③ Separated/Divorced ④ Widowed

4. What is your highest level of education?

① No formal education ② Elementary school graduate ③ Middle school graduate ④ High school graduate ⑤ college graduate

5. What is your religion?

① Yes (Specify: __________) ② No

6. Do you have any current medical conditions?

① Yes (Specify: __________) ② No

## Korean Mini-Mental State Examination 2nd Edition (K-MMSE-2)

## Registration (3 points)

Airplane: 0 1

Pencil: 0 1

Pine tree: 0 1

## Orientation - Time (5 points)

Year: 0 1

Month: 0 1

Day: 0 1

Day of the week: 0 1

Season: 0 1

## Orientation - Place (5 points)

Country: 0 1

City/Province: 0 1

Type of place (e.g., hospital, home, etc.): 0 1

Current location name: 0 1

Floor number: 0 1

## Recall (3 points)

Airplane: 0 1

Pencil: 0 1

Pine tree: 0 1

## Attention and Calculation (5 points)

100 - 7: 0 1

- 7: 0 1

- 7: 0 1

- 7: 0 1

- 7: 0 1

## Language and Visuospatial Ability (9 points)

Naming Objects: eye, ears 0 1

Repetition: Say the phrase "A picture is worth a thousand words": 0 1 2

Understanding: Point to the circle, then point to the square, and finally point to the triangle : 0 1 2 3

Reading: (close your eyes) 0 1

Writing: Write a sentence about today's weather: 0 1

**Drawing**

Copying the pentagon: 0 1

## Total Score: /30

##
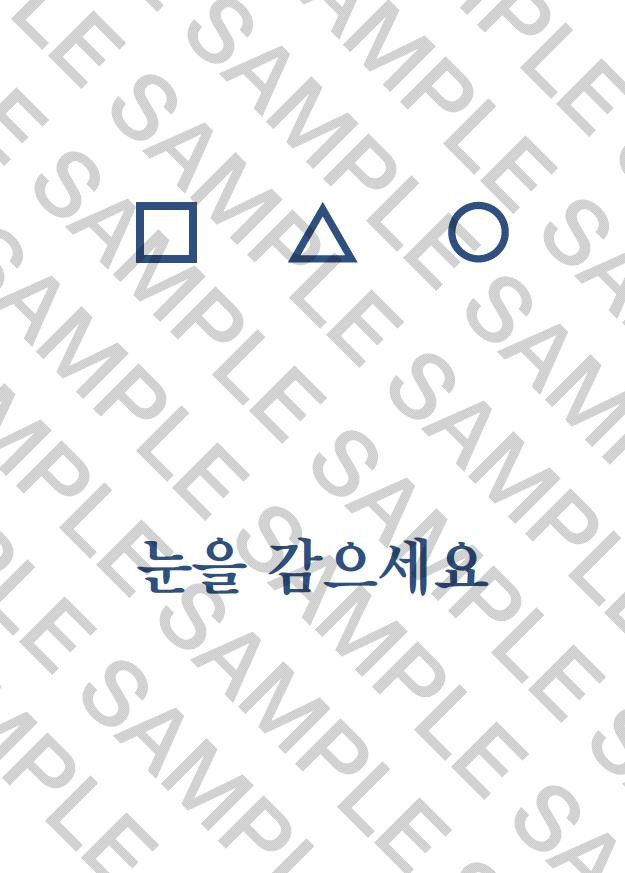


CLOSE YOUR EYES

##
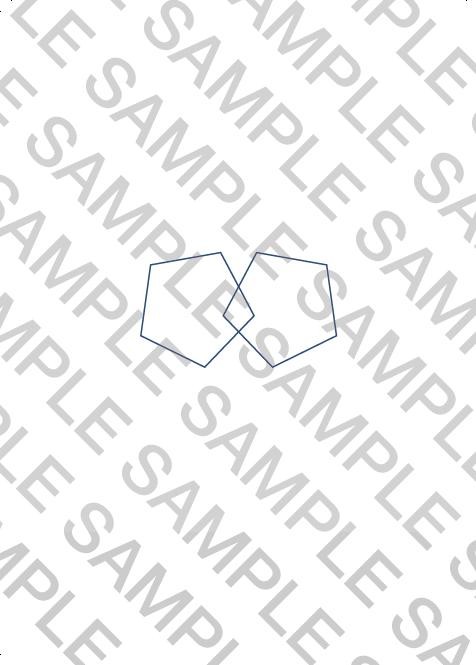


## Korean Geriatric Depression Scale - Short Form (K-GDS)

Please answer based on how you have felt over the past week.

1. Are you generally satisfied with your life? ① Yes ② No

2. Have you experienced a significant decline in activities and interests?* ① Yes ② No

3. Do you feel hopeful about the future? ① Yes ② No

4. Do you spend most of your time with a clear mind? ① Yes ② No

5. Do you feel happy most of the time? ① Yes ② No

6. Do you think that being alive is a beautiful thing? ① Yes ② No

7. Do you sometimes feel discouraged and depressed?* ① Yes ② No

8. Do you feel that your life is worthless?* ① Yes ② No

9. Do you find life very interesting? ① Yes ② No

10. Do you feel full of energy? ① Yes ② No

11. Do you often feel emotionally disturbed by trivial matters?* ① Yes ② No

12. Do you often feel like crying?* ① Yes ② No

13. Do you enjoy waking up in the morning? ① Yes ② No

14. Do you find it easy to make decisions? ① Yes ② No

15. Do you feel as comfortable as you used to? ① Yes ② No
