# Supplementary material for: Social robot PIO intervention for improving cognitive function and depression in older adults with mild to moderate dementia in day care centers: A randomized controlled trial
Source: PLoS One. 2025 Apr 22;20(4):e0321745. doi: 10.1371/journal.pone.0321745 (PMC12013943; doi:10.1371/journal.pone.0321745)
Supplement: S4 File — (PDF) [file pone.0321745.s004.pdf]

연구계획서(인간대상연구)

1. 기본 사항

**연구과제명**	국문	소셜로봇을 활용한 인지기반중재 프로그램이 노인의 인지기능, 우울, 삶의 질에 미치는 효과		
	영문	The effects of cognitive-based intervention using social robot on cognitive function, depression, quality of life of the elderly		
**책임연구자**	성명	오혜경	전공분야	간호학
	소속	대구대학교 간호대학	간호학과	
**공동연구자**	성명	임준서	직위	연구원
	소속	서울대학교 간호대학	전공분야	간호학
	전화	-	휴대전화	
	E-mail	junseo@snu.ac.kr	Fax	-
**연구관련자 (보조원 등)**	성명		직위	
	소속		전공분야	
	전화		휴대전화	
	E-mail		Fax	
**의뢰자 (또는 의뢰기관)**	의뢰자명			
	주소			
	전화		휴대전화	
**연구비 지원기관 (해당 경우)**	기관명			
	주소			
	대표전화		대표 휴대전화	

2. 연구의 목적 및 배경

**연구 목적**	본 연구의 목적은 소셜로봇을 활용한 인지기반중재 프로그램이 노인의 인지기능, 우울, 삶의 질에 미치는 효과를 확인하기 위해서입니다.
**연구 배경**	전 세계 65세 이상 인구는 2020년 9.3%에서 2050년 약 16%로 증가할 것으로 예상된다(United Nations, 2020). 우리나라 65세 이상 노인인구는 2020년 15.7%로 고령사회에 속해 있다(통계청, 2021). 노화로 인한 인지기능의 저하는 개인의 적응 능력을 감소시키고, 우울과 불안 같은 정서적 문제를 야기할 뿐만 아니라 사회적으로 대인 관계를 맺기도 어려워져 삶의 질이 떨어진다(Zelinski & Gilewski, 2004). 인지기능은 학습 및 기억 능력, 주의 집중력, 언어능력, 운동능력, 시각 및 공간 지각능력, 전두염 관리기능, 성격 및 정서기능(김혜순 et al., 2010)으로 노인의 인지기능이 높을수록 우울 정 도가 낮게 나타나고(손의성 & 김동배, 2005), 인지기능이 저하된 노인은 우울 증상이 증가하여 삶의 질이 저하되는 요인으로 작용한다(김혜순 et al., 2010).<br>최근 소셜 로봇(Social robot)을 활용한 중재 연구가 세계적으로 다양하게 시도되고 있다(오진환, 2018). 국내 사례를 보면 구로구에 거주하는 65세 이상 169명의 노인을 대상으로 소셜 로봇 효돌을 활용하여 대상자와 소셜 로봇과의 접촉과 동작 등에 대해 반응하는 센서를 기반으로 작동하여 상호작용을 통한 정서교감이 이루어지도록 하였다(김선화 et al., 2020). 외국의 경우 정서적 안정과 상호작용 촉진을 위해 물개 형태의 소셜 로봇 PARO를 치매 노인들에게 적용한 연구가 주로 시행되었다(Leng et al., 2019; Park et al., 2020).<br>이렇듯 국내외적으로 노인 대상 소셜 로봇을 활용한 연구가 이루어지고 있지만, 어떠한 형태의 로봇이 노인들의 인지기능과 불안, 우울 등 정서적 부분에 일관적인 효과를 보이는지에 대해서는 아직 밝혀지지 않고 있다. 따라서 본 연구는 노인의 인지기능을 향상시킬 수 있도록 다 영역을 자극할 수 있는 소셜 로봇을 활용한 인지 기반 중재 프로그램을 적용하여 노인들의 인지능력을 증가시키고, 우울을 감소시켜 삶의 질을 증가시키는 것을 목적으로 그 효과를 확인하고자 한다.

3. 연구 기간

**예상 연구기간**	**IRB 승인 이후** ~ 2023년 12월 31일

4. 연구방법

① 연구방법 개요

본 연구는 대구지역 거주 노인 대상 소셜로봇을 활용한 인지기반중재 프로그램이 노인의 인지기능, 우울, 삶의 질에 미치는 효과에 관한 연구입니다. 연구는 연구대상자를 모집한 대구지역 주간보호시 설 또는 재활병원에서 시행될 예정으로 기관을 통해 참여하기를 원하는 대상자에 한해 진행됩니다. 연구참여자는 실험군과 대조군으로 나뉘어 PIO 로봇 프로그램 12회기와 함께 프로그램 시작 전, 후 에 연구 설문측정을 2회 실시하게 됩니다. 실험군에게 PIO 로봇 프로그램에 12회기(주당 2회기, 1회 기당 50분 가량, 대상자 1명당 로봇 1대가 주어지며, 진행자가 메인보드에서 전체 로봇을 조정하면서 프로그램을 수행함. 환자 안전과 원활한 프로그램 진행을 위해 보조진행자 1인과 함께 진행 예정, 1회당 10명 이내로 진행할 예정임), 대조군에는 사전, 사후 설문지 이외 제공되는 프로그램은 없습니다. 단, 대조군의 경우, 본인이 동일한 프로그램에 참여하기를 원하는 분에 한해, 실험군의 12회기 프로그램이 끝난 후 약 2주 후에 동일한 로봇 프로그램 12회기를 받을 수 있습니다.

1) 사용하는 측정도구

(1) 인지기능: 본 연구에서는 K-MMSE-2: SV (Standard version)를 사용하여 기억등록, 시간 지남력, 기억회상, 주의집중 및 계산, 언어, 그리기로 구성된 도구를 사용하여 측정한 점수를 의미한다.

(2) 우울: Sheikh와 Yesavage (1986)가 개발한 노인 우울척도 단축형을 기백석(1996)이 우리나라에 맞도록 수정한 한국형 노인 우울 간이척도(Geriatric Depression Scale Short Form Korea Version, GDSSF-K)를 사용하여 측정한 점수를 의미한다.

2) PIO 로봇에 대한 설명 및 안정성:

-로봇 활용에 대한 설명: 본 프로그램에서 사용될 PIO 로봇(10대)은 책임연구자가 ㈜ 와이닷츠에 비용을 지불하고 대여하여 연구를 수행할 예정임.

(1) 1회기: 소셜로봇 피오를 알에서부터 부화시켜 성장시켜 가는 이야기가 주제로 태블릿 pc 화면에 알이 등장하고 이 알을 부화시키기 위해 대상자는 화면에 지시된 방향에 맞추어 일정하게 흔들게 되 며, 알에 금이 가면서 소셜로봇이 부화하게 된다.

(2) 2회기: 아기 소셜 로봇 꾸미기로 태블릿 pc에 나타난 로봇의 둥지와 배경을 선택하고 손가락으로 선택하여 색칠한다.

(3) 3회기: 우유 먹이고 재우기로 음성과 자막에 따라 젖병에 물을 채우고 숟가락을 여러번 눌러 분 유를 채운 후 태블릿 pc를 흔들어 우유를 준비하고 완료되면 로봇이 우유를 마시고 활동이 종료된 다.

(4) 4회기: 소셜 로봇 옷 만들기로 태블릿 pc에 표시된 패턴을 선택하고 정해진 위치에 배역하고 색 칠하게 한다.

(5) 5회기: 말 가르치기로 구조화된 상황에 따라 적절한 응답을 학습한 소셜로봇은 이제부터 말을 할 수 있게 된다.

(6) 6회기: 동작과 체조 가르치기로 소셜 로봇 이마에 있는 카메라에 파란 카드를 보여주면 소셜 로 봇이 파란색 카드를 인식하여 소셜 로봇의 머리가 카드의 움직이는 방향에 맞추어 움직이게 된다.

(7) 7회기: 애벌레 잡기로 푸른 이파리 위에 애벌레를 터치하여 잡게 되는데 독이 있는 붉은 색 애벌 레를 추가하여 이를 터치하였을 때 획득한 애벌레 수에서 차감하는 방식으로 진행한다.

(8) 8회기: 대왕 애벌레 잡기로 애벌레의 몸통을 누르면 맨 끝 부분의 녹색 동그라미가 사라지게 되고 계속 눌러 녹색 동그라미가 모두 사라지면 대왕 애벌레 1마리를 획득하게 된다.

(9) 9회기: 색깔 가르치기로 태블릿 pc에 5가지 색깔 버튼이 나오고 선택한 색과 연관된 콘텐츠가 진행된다.

(10) 10회기: 소셜 로봇과 장보기로 로봇이 필요하다고 알려주는 쇼핑 목록을 듣고 기억한 후 슈퍼에서 물건을 선택한다.

(11) 11회기: 소셜 로봇과 노래하고 초상화 그리기로 로봇이 박수를 치면 대상자도 함께 박수를 치고 로봇의 움직임을 인지하고 따라할 수 있도록 구성하였으며, 색깔 팔레트의 색을 선택하여 소셜 로봇과 선택한 배경을 색깔하게 한다.

(12) 12회기: 작별로 정서적 교감을 목표로 하였다. 프로그램을 마치며 소감을 나누고 소셜 로봇과 이별 인사를 하고 전체 프로그램을 마무리한다.

-로봇 안정성: 로봇을 만지고 활용 시, 안전사고가 일어나지 않도록 대부분 곡선으로 구성되어 있으며, 태블릿 pc의 화면을 보고 따라하는 위주의 프로그램으로 안정성에는 특별한 문제가 없습니다.

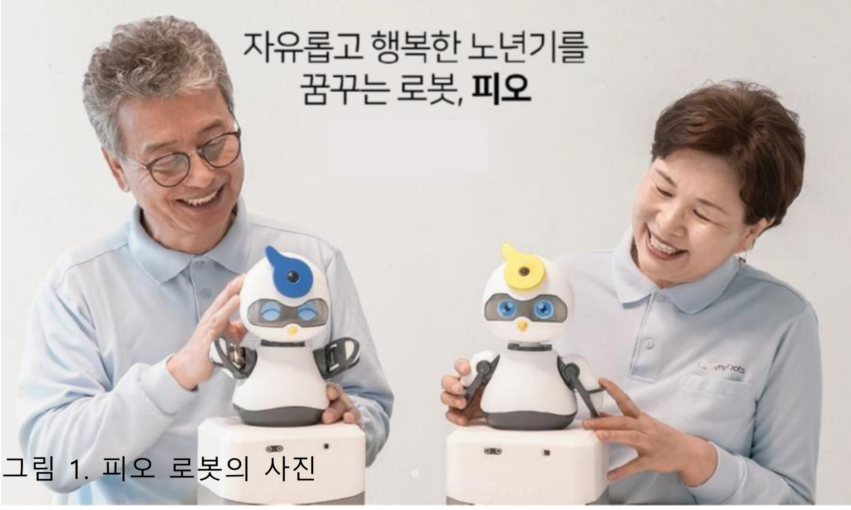

그림 1. 피오 로봇의 사진

② 연구대상자 모집 방법

**연구대상자 모집방법**	대구지역 주간보호시설 또는 재활병원 2곳의 협조를 구해 연구에 참여하기를 원하는 대상자를 모집할 계획입니다.
**연구대상자의 동의 취득 절차**	실험연구 시작 전 대상자에게 연구설명서 및 동의서에 대한 내용을 충분히 설명한 후 대상자에게 서면 동의를 득할 예정입니다.

③ 연구대상자의 선정기준 및 제외기준

**연구대상자 선정 기준**	1) 연구목적을 이해하고 설문에 동의한 만 65세 이상 노인<br>2) 국문해독이 가능하고 경중등도 이하(Mild~Moderate) 인지장애가 있어 의사소통이 가능한 노인
**연구대상자 제외 기준**	1) 정신분열증, 조울증과 같은 정신과적 병력이 있는 노인<br>2) 중등도 이상의 인지장애가 있어 의사소통이 불가능한 노인

④ 목표 연구대상자의 수 및 산출근거

**연구대상자 수**	약 80명
**연구대상자 산정근거**	연구대상자 수는 G power 3.1.9.6 program(Faul, Erdfelder, Buchner, & Lang, 2009)을 이용하여 두 집단의 독립표본 양측 t-검정을 위해 효과크기 0.7, 유의수준 .05, 검정력 0.8로 하였을 때 요구되는 최소표본은 각 군당 34명으로 총 68명이다. 이에 탈락률을 고려하여 고려하여 총 80명을 선정한다. 효과크기는 노인대상 우울 프로그램 효과성에 대한 메타분석을 시행한 선행연구(김일식, 2015)에서 효과크기 0.7로 나타난 것을 근거로 하였다.

⑤ 연구 설계**(해당되는 경우)**

**비교군 설정**	본 연구 동안 새로운 인지기능 향상 프로그램이나 다른 그룹 활동에 신규로 참여하지 않은 참가군이다.
**무작위 배정**	연구대상자 기준에 적합한 대상자를 웹사이트 (http://www.randomization.com)에서 제공되는 프로그램을 이용하여 실험군과 대조군으로 무작위 배정하였으며, 배정표는 연구의 수행과 관련 없는 제3자가 불투명한 봉투에 차폐하여 관리할 예정이다.
**눈가림법**	연구는 이중맹검으로 시행한다. 참가자들은 실험군과 대조군 중 어느 그룹에 속하는지 알지 못하며, 데이터 수집은 실험군과 대조군을 알지 못하는 연구 보조원이 수집할 것이다. 데이터 분석은 객관적인 결과를 위해 통계분석가에 의해 수행될 것이다.

⑥ 임상연구 경우의 사항**(해당되는 경우)**

**투여량, 투여방법, 투여기간 및 설정 사유**	
**대조약 사용시 대조약 및 사유**	
**관찰항목, 관찰검사방법 및 임상검사항목**	
**효과평가변수, 평가방법 및 해석 방법**	
**안전성 평가기준, 평가방법 및 보고방법**	

⑦ 통계 원칙 및 방법

**통계분석 원칙**	본 연구에서는 소셜로봇을 활용한 인지기반중재 프로그램이 인지기능, 우울, 삶의 질에 미치는 영향을 확인하기 위해 본 연구의 수집된 자료를 이용하게 된다.

**통계분석 방법**	1) 연구 참여자의 일반적 특성은 빈도, 백분율, 평균, 표준편차로 산출하여 분석할 예정이다.<br>2) 집단 간 동질성 검증은 t-test, χ2 test, Fisher’s exact test를 이용하여 분석할 예정이다.<br>3) 프로그램의 효과를 확인하기 위한 가설 검정은 정규분포일 경우 t-test, 비정규분포일 경우 Mann-Whitney U test로 분석할 예정이다.<br>4) 도구의 신뢰도 검증은 크론바흐의 알파(Cronbach's Alpha) 계수로 분석할 예정이다.

⑧ 설문연구의 경우 설문지 사후 처리 방법**(연구방법이 설문조사의 경우 필수기재)**

**설문지 활용방법**	본 연구의 설문지 결과는 소셜로봇을 활용한 인지기반중재 프로그램이 인지기능, 우울, 삶의 질에 미치는 영향을 확인하기 위한 연구에 활용될 예정이다.
**설문지 보관기간 및 방법**	본 설문지는 연구 종료시점으로부터 3년간 보관할 예정이며, 보관방법은 연구책임자(오혜경)와 외부연구원(임준서)만 접근이 허락되며, 설문자료는 개인연구실 내 사물함에 이중 시건장치를 한 방법으로 보관이 될 것이다.
**설문지 폐기시기 및 방법**	설문지 폐기 시기는 연구 종료시점으로부터 3년간 보관 이후 바로 파쇄하여 폐기할 예정이며, 컴퓨터 상의 연구결과 또한 암호화하여 보관 뒤 파일 영구삭제 예정이다.

5. 연구대상자의 안정성(모든 항목 상세 기재 요망)

**예측효과**	본 연구를 통해 대상자는 인지기능, 우울, 삶의 질 정도가 개선되는 효과를 가지게 되고 이후 소셜로봇을 위한 연구를 적용하는 데 기초자료가 될 수 있는 간접적인 효과가 있다.
**예측 부작용 및 위험성**	자발적으로 참여하기를 원하는 대상자를 선정하여 프로그램을 진행할 예정이므로 큰 문제 소지는 없을 것으로 예상되나, 노인분들의 건강상태를 잘 살피면서 프로그램을 진행할 예정이다.
**연구대상자 보상 규약 및 이익**	본 연구의 부작용이나 위험요소는 없지만 12회기의 프로그램 참여 및 실험 전‧후 15~20분 가량의 설문지 응답으로 인한 번거로움이 발생할 수 있으므로 실험후, 설문연구에 참여 시, 연구대상자에게 감사의 뜻으로

	3000~4000원 정도 되는 작은 답례품이 증정될 예정이다.
**연구대상자 안정보호에 대한 대책**	참여대상자 중 건강상의 문제나 불편감을 표현할 경우 즉시 프로그램에서 제외하고 건강상의 문제일 경우, 관련 의료기관에 연계를 의뢰할 예정이다. 또한, 연구대상자가 원한다면 언제든지 연구를 중단할 수 있다는 사실을 미리 설명할 예정이다.
**연구대상자의 개인정보 범위 및 관리 대책**	본 연구의 참여로 수집되는 개인정보의 범위는 연령, 성별, 결혼상태, 자녀유무, 반려동물 부양여부, 학력, 종교, 현재 가지고 있는 질환, 신체적 장애, 수급 대상자 여부이다. 이러한 정보는 개인정보관리책임자(연구책임자: 오혜경)에게만 접근이 허락되며, 개인 연구실 내 사물함에 이중 시건장치를 한 방법으로 보관이 될 예정이며, 개인정보를 포함한 연구결과 정리양식은 연구책임자의 컴퓨터에 비밀번호를 부여하여 보관할 계획이다. 연구를 위해 수집된 자료는 연구 종료 후 3년간 보관한후 폐기할 예정이다.

6. 참고문헌

-기백석. (1996). 한국판 노인 우울 척도 단축형의 표준화 예비연구. 신경정신의학, 35(2), 298-306.

-김선화, 김지희, 김태환, 이동림, 최선용, 이호상, . . . 남일성. (2020). 한국형 소셜로봇 효돌이 지역사회 거주 독거노인의 우울증상과 삶의 질에 미치는 영향. 한국노년학, 40(5), 1021-1034.

-김일식, 신혜숙, & 서호찬. (2015). 노인대상 우울 프로그램 효과성에 대한 메타분석. 한국노년학, 35(4), 1061-1079.

-김혜순, 이여진, 박광희, 강운구, & 이병문. (2010). 인지건강증진 프로그램이 노인의 인지기능, 우울 및 삶의 질에 미치는 효과. The Journal of the Korea Contents Association, 10(8), 227-239.

-손의성, & 김동배. (2005). 한국노인의 우울 관련변인에 관한 메타분석. 한국노년학, 25(4), 167-187.

-오진환. (2018). 로봇 테크놀로지 기반의 노인간호 활용전망. 노인간호학회지, 50, 127-136.

-통계청. (2021). 2021 고령자 통계. 사회통계기획과. Retrieved from https://kostat.go.kr/portal/korea/kor_nw/1/1/index.board?bmode=read&aSeq=403253.

-Kim SH, Jo MW, Ahn J, Shin S, Ock M, Park J, Luo N. The Valuation of EQ-5D-5L Health States in Korea. Value Health. 2014 Nov;17(7):A753. doi: 10.1016/j.jval.2014.08.211. Epub 2014 Oct 26. PMID: 27202736.

-Leng, M., Liu, P., Zhang, P., Hu, M., Zhou, H., Li, G., . . . Chen, L. (2019). Pet robot intervention for people with dementia: A systematic review and meta-analysis of randomized controlled

trials. Psychiatry Res, 271, 516-525.

-Park, S., Bak, A., Kim, S., Nam, Y., Kim, H. S., Yoo, D.-H., & Moon, M. (2020). Animal-assisted and pet-robot interventions for ameliorating behavioral and psychological symptoms of dementia: A systematic review and meta-analysis. Biomedicines, 8(6), 150.

-Sheikh, J. I., & Yesavage, J. A. (1986). Geriatric Depression Scale (GDS): recent evidence and development of a shorter version. Clinical Gerontologist: The J ournal of Aging and Mental Health.

-UN. (2020). World Population Ageing 2020 Report. Department of Economic and Social Affairs. Retrieved from https://www.un.org/development/desa/pd/sites/www.un.org.development.desa.pd/files/undesa_pd-2020_world_population_ageing_highlights.

-Zelinski, E. M., & Gilewski, M. J. (2004). A 10-item Rasch modeled memory self-efficacy scale. Aging Ment Health, 8(4), 293-306.

대구대학교 생명윤리위원회 위원장 귀하

대구대학교
DAEGU UNIVERSITY

Research Plan (Human Subjects Research)

1. Generals

**Research Project Name**	Korean	소셜로봇을 활용한 인지기반중재 프로그램이 노인의 인지기능, 우울, 삶의 질에 미치는 효과		
	English	The effects of cognitive-based intervention using social robot on cognitive function, depression, quality of life of the elderly		
**Principal Researcher**	Name	Oh Hye-Kyung	Field of Study	Nursing
	Affiliation	Daegu University College of Nursing	Department of Nursing	
**Joint Researcher**	Name	Lim JunSeo	Position	Researcher
	Affiliation	Seoul National University College of Nursing	Field of Study	Nursing
	Phone	-	Cell-Phone	
	E-mail	junseo@snu.ac.kr	Fax	-
**Research Related Person (assistant, etc.)**	Name		Position	
	Affiliation		Field of Study	
	Phone		Cell-Phone	
	E-mail		Fax	
**Requester (or Request Branch)**	Requester			
	Address			
	Phone		Cell-Phone	
**Research Funding Agency (In that case)**	Organization name			
	Address			
	Main Phone		Cell-Phone	

2. Purpose and background of the study

**Purpose**	The purpose of this study is to determine the effects of a cognitive-based intervention program using social robots on the cognitive function, depression, and quality of life of the elderly.
**Background**	The global population aged 65 or older is expected to increase from 9.3% in 2020 to approximately 16% in 2050 (United Nations, 2020). Korea's elderly population aged 65 or older is 15.7% in 2020, belonging to an aging society (Statistics Korea, 2021). Deterioration of cognitive function due to

	aging reduces an individual's ability to adapt, causes emotional problems such as depression and anxiety, and also makes it difficult to form interpersonal relationships, reducing the quality of life (Zelinski & Gilewski, 2004). Cognitive functions include learning and memory ability, attention concentration, language ability, motor ability, visual and spatial perception ability, frontalitis management function, personality, and emotional function (Kim Hye-soon et al., 2010). The higher the cognitive function of the elderly, the higher the level of depression. appears to be low (Son & Kim, 2005), and in elderly people with reduced cognitive function, depressive symptoms increase, which acts as a factor in lowering quality of life (Kim et al., 2010).<br>Recently, various intervention studies using social robots have been attempted around the world (Oh, 2018). In a domestic case, the social robot Hyodol was used for 169 seniors aged 65 or older living in Guro-gu, and emotional connection was achieved through interaction by operating based on sensors that react to the subject's contact and movement with the social robot. (Kim et al., 2020). In foreign countries, studies were mainly conducted on applying PARO, a seal-shaped social robot, to elderly people with dementia to promote emotional stability and interaction (Leng et al., 2019; Park et al., 2020).<br>Although research using social robots for the elderly is being conducted both domestically and internationally, it is not yet clear what type of robot has a consistent effect on the cognitive function and emotional aspects such as anxiety and depression of the elderly. Therefore, this study applied a cognitive-based intervention program using a social robot that can stimulate multiple areas to improve the cognitive function of the elderly, reduce depression, and increase quality of life. The purpose is to check the effect.<br>A decline in cognitive function due to aging reduces individual adaptability, causes emotional problems such as depression and anxiety, and also makes it difficult to have interpersonal relationships, resulting in poor quality of life (Zelinski & Gilewski, 2004). Cognitive function means the learning and memory, attention, language, motor, visual and spatial perception, personality and emotional function (Kim Hye-soon et al., 2010). The higher the cognitive function related to lower degree of depression (Hand & Kim Dong-bae, 2005). The lower the cognitive function related to lower the quality of life (Kim Hye-soon et al., 2010).<br>Recently, various intervention studies using social robots have been attempted worldwide (Oh, 2018). In South Korea, 169 elderly people (aged 65 or older) living in Guro-gu were operated based on sensors that respond to contact and movement between the target and social robots to achieve emotional communication through interaction (Kim et al., 2020). In abroad, studies have mainly been conducted to apply seal-type social robot (PARO) to promote emotional stability and interaction in to the dementia elderly (Leng et al., 2019; Park et al., 2020).<br>Although research using social robots for the elderly is being conducted, it is not yet known which type of robot has a consistent effect on the cognitive function, anxiety, and depression of the elderly. Therefore, this study aims to confirm the effect of applying a cognitive-based intervention program using social robots that can stimulate multiple areas to improve the cognitive function of the elderly, thereby increasing the cognitive ability of the elderly, reducing depression, and increasing the quality of life.

3. Study periods

Expected research period	IRB After approval ~ 31.12.2023

4. Research methods

① Overview of research methods

This study is about the effects of a cognitive-based intervention program using social robots on the cognitive function, depression, and quality of life of the elderly living in the Daegu area. The study will be conducted at a day care facility or rehabilitation hospital in the Daegu area where research subjects were recruited, and will only be conducted for those who wish to participate through the institution. Research participants will be divided into an experimental group and a control group and will undergo 12 sessions of the PIO robot program and conduct research questionnaires twice before and after the start of the program. The experimental group is given 12 sessions of the PIO robot program (2 sessions per week, about 50 minutes per session, 1 robot per subject, and the facilitator performs the program while controlling the entire robot on the main board. To ensure patient safety and smooth program progress. For this reason, it will be conducted with one assistant facilitator and will be conducted with less than 10 people per session), and no program will be provided to the control group other than pre- and post-questionnaires. However, in the case of the control group, only those who wish to participate in the same program can receive 12 sessions of the same robot program approximately 2 weeks after the end of the 12-session program of the experimental group.

1) Measurements

(1) Cognitive function: In this study, K-MMSE-2: SV (Standard version) was used to measure scores using tools consisting of memory registration, temporal orientation, memory recall, attention and calculation, language, and drawing. it means.

(2) Depression: Measured using the Geriatric Depression Scale Short Form Korea Version (GDSSF-K), a short form of the Geriatric Depression Scale developed by Sheikh and Yesavage (1986) modified for Korea by Baekseok Ki (1996). It means score.

2) Description and stability of PIO robot:

- Explanation on robot use: The lead researcher plans to rent the PIO robots (10 units) to be used in this program at a cost to WhyDots Co., Ltd. to conduct research.

(1) Session 1: The theme is the story of hatching and growing the social robot Pio from an egg. An egg appears on the tablet PC screen, and in order to hatch the egg, the subject shakes it steadily in the direction indicated on the screen, and the egg When a crack occurs, a social robot hatch.

(2) Session 2: To decorate the baby social robot, select the robot's nest and background displayed on the tablet PC and

select and color it with your finger.

(3) Session 3: Feed milk and put it to sleep. Fill the baby bottle with water according to the voice and subtitles, press the spoon several times to fill with powdered milk, shake the tablet PC to prepare milk, and when completed, the robot drinks the milk and the activity ends.

(4) Session 4: Making social robot clothes involves selecting a pattern displayed on a tablet PC, assigning it to a designated location, and coloring it.

(5) Session 5: Teaching speech the social robot, which has learned appropriate responses according to structured situations, can now speak.

(6) Session 6: Teaching movement and gymnastics. When you show a blue card to the camera on the social robot's forehead, the social robot recognizes the blue card and the social robot's head moves in accordance with the direction of the card.

(7) Session 7: Catching caterpillars involves catching caterpillars by touching them on green leaves. A poisonous red caterpillar is added and deducted from the number of caterpillars obtained when touched.

(8) Session 8: When you press the body of the caterpillar to catch the giant caterpillar, the green circle at the end will disappear. If you continue to press until all the green circles disappear, you will obtain one giant caterpillar.

(9) Session 9: Teaching colors. Five color buttons appear on the tablet PC, and content related to the selected color is played.

(10) Session 10: Social robot and grocery shopping. After hearing and remembering the shopping list that the robot tells you need, you select items from the supermarket.

(11) Session 11: Singing with a social robot and drawing a portrait. When the robot claps, the subject also claps together and is able to recognize and follow the robot's movements. By selecting a color from the color palette, the social robot and the selected background are created. Color it.

(12) Session 12: Farewell, aimed at emotional connection. At the end of the program, we share our thoughts, say goodbye to the social robot, and conclude the entire program.

-Robot stability: Most of the robots are curved to prevent safety accidents when touching and using them, and there are no particular problems with stability as it is a program that focuses on watching and following the screen on a tablet PC.

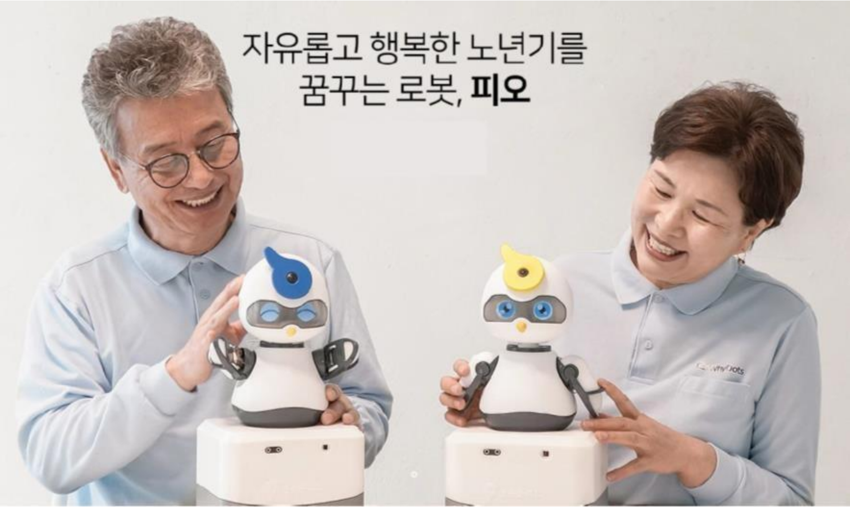

Figure 1. Photo of Pio Robot

② Recruiting

**Recruitment method**	We plan to recruit subjects who wish to participate in the study with the cooperation of two day care facilities or rehabilitation hospitals in the Daegu area.
**Procedure for obtaining consent from research subjects**	Before starting the experimental study, the contents of the research description and consent form will be fully explained to the subjects, and then written consent will be obtained from the subjects.

③ Selection criteria and exclusion criteria

**Selection criteria**	1) Seniors aged 65 years or older who understand the purpose of the study and agree to the survey<br>2) Elderly people who can decipher Korean and communicate due to mild to moderate cognitive impairment
**Exclusion criteria**	1) Elderly people with a psychiatric history such as schizophrenia or bipolar disorder<br>2) Elderly people who are unable to communicate due to moderate or severe cognitive impairment

④ Number of target research subjects and calculation basis

**Number of research subjects**	About 80 people
**Calculation of research subjects**	The number of study subjects was set to an effect size of 0.7, significance level of .05, and power of 0.8 for a two-tailed t-test of independent samples of two groups using the G power 3.1.9.6 program (Faul, Erdfelder, Buchner, & Lang, 2009). The minimum sample required is 34 people in each county, for a total of 68 people. Accordingly, taking into account the dropout rate, total of 80 people were selected. The effect size was based on a previous study that conducted a meta-analysis on the effectiveness of depression programs for the elderly (Kim, 2015), which found an effect size of 0.7.

⑤ Study design (if applicable)

**Comparison group settings**	This group of participants did not participate in any new cognitive function improvement programs or other group activities during this study.

**Random assignment**	Subjects who met the research subject criteria were randomly assigned to the experimental group and control group using a program provided on the website (http://www.randomization.com), and the allocation table was placed in an opaque envelope by a third party unrelated to the conduct of the study. It will be managed by shielding.
**Blinding**	It will be conducted in a double-blind manner. Participants do not know which group they belong to: the experimental group or the control group. Data collection will be conducted by research assistants who are unaware of the experimental and control groups. Data analysis was performed by a statistical analyst to ensure objective results.

⑥ Clinical trials (if applicable)

**Dosage, administration method, administration period, and reason for setting**	
**Reference drug and reasons for using reference drug**	
**Observation items, observation test methods, and clinical test items**	
**Effectiveness evaluation variables, evaluation methods and interpretation methods**	
**Safety evaluation criteria, evaluation methods and reporting methods**	

⑦ Statistical Principles and Methods

**Statistical Analysis Principles**	In this study, the data collected in this study will be used to determine the impact of a cognitive-based intervention program using social robots on cognitive function, depression, and quality of life.

**Statistical analysis method**	1) The general characteristics of research participants will be calculated and analyzed in terms of frequency, percentage, average, and standard deviation.<br>2) Verification of homogeneity between groups will be analyzed using t-test, $\chi 2$ test, and Fisher’s exact test.<br>3) The hypothesis test to confirm the effectiveness of the program will be analyzed by t-test in case of normal distribution and Mann-Whitney U test in case of non-normal distribution.<br>4) The reliability of the tool will be verified using Cronbach's Alpha coefficient.

⑧ Survey Research, Questionnaire Post-processing Method (required information if the research method is a survey)

**How to use the questionnaire**	The results of this study's questionnaire will be used in research to determine the impact of cognitive-based intervention programs using social robots on cognitive function, depression, and quality of life.
**Questionnaire storage period and method**	This questionnaire will be stored for 3 years from the end of the study. As for the storage method, only the research director (Oh Hye-kyung) and external researcher (Lim Jun-seo) will be allowed access, and the survey data will be stored in a locker in the personal laboratory with a double lock device.
**When and how to dispose of questionnaires**	The questionnaire will be shredded and discarded immediately after being stored for 3 years from the end of the study, and the research results on the computer will also be encrypted and permanently deleted after storage.

5. Stability of Research Subjects (all items must be filled out in detail)

**Predicted Effect**	Through this study, subjects' cognitive function, depression, and quality of life are improved, and there is an indirect effect that can serve as basic data for applying research on social robots in the future.
**Predicted side effects and risks**	Since the program will be carried out by selecting those who want to voluntarily participate, it is not expected that there will be any major problems, but the program will be carried out while carefully monitoring the health status of the elderly.
**Research subject compensation regulations and benefits**	Although there are no side effects or risk factors in this study, inconveniences may arise due to participation in the 12-session program and answering questionnaires for approximately 15 to 20 minutes before and after the experiment. Therefore, we express our gratitude to the research subjects when participating in the survey study after the experiment. A small gift worth about 3,000 to 4,000 won will be given as a gift.

**Measures to protect the safety of research subjects**	If any of the participants expresses health problems or discomfort, they will be immediately excluded from the program, and if it is a health problem, they will be referred to a relevant medical institution. Additionally, it will be explained in advance that research subjects can stop the research at any time if they wish.
**Scope and management measures of personal information of research subjects**	The scope of personal information collected through participation in this study includes age, gender, marital status, presence of children, whether or not you support a pet, education level, religion, current disease, physical disability, and eligibility for benefits. Access to this information is permitted only to the person in charge of personal information management (research director: Hye-kyung Oh), and it will be stored in a locker in the personal laboratory using a double-lock device. The research results organization form, including personal information, will be stored on the researcher's computer. We plan to give it a password and store it. The data collected for the study will be stored for three years after the end of the study and then destroyed.

6. References

- Ki Baek-seok. (1996). Preliminary study on standardization of the short version of the Korean version of the Elderly Depression Scale. Neuropsychiatry, 35(2), 298-306.

-Kim Seon-hwa, Kim Ji-hee, Kim Tae-hwan, Lee Dong-rim, Choi Seon-yong, Lee Ho-sang, . . . Nam Il-seong. (2020). The impact of Hyodol, a Korean social robot, on depressive symptoms and quality of life in elderly people living alone in the community. Korean Gerontology, 40(5), 1021-1034.

-Kim Il-sik, Shin Hye-sook, & Seo Ho-chan. (2015). Meta-analysis on the effectiveness of depression programs for the elderly. Korean Gerontology, 35(4), 1061-1079.

-Hye-sun Kim, Yeo-jin Lee, Gwang-hee Park, Un-gu Kang, & Byeong-moon Lee. (2010). Effects of cognitive health promotion programs on cognitive function, depression, and quality of life in the elderly. The Journal of the Korea Contents Association, 10(8), 227-239.

-Son Eui-seong, & Kim Dong-bae. (2005). Meta-analysis on variables related to depression in Korean elderly. Korean Gerontology, 25(4), 167-187.

-Oh Jin-hwan. (2018). Prospects for the use of elderly care based on robot technology. Journal of Geriatric Nursing, 50, 127-136.

-Statistical Office. (2021). 2021 Senior Citizen Statistics. Social Statistics Planning Department. Retrieved from https://kostat.go.kr/portal/korea/kor_nw/1/1/index.board?bmode=read&aSeq=403253.

-Kim SH, Jo MW, Ahn J, Shin S, Ock M, Park J, Luo N. The Valuation of EQ-5D-5L Health States in Korea. Value Health. 2014 Nov;17(7):A753. doi: 10.1016/j.jval.2014.08.211. Epub 2014 Oct 26. PMID: 27202736.

-Leng, M., Liu, P., Zhang, P., Hu, M., Zhou, H., Li, G., . . . Chen, L. (2019). Pet robot intervention for people with dementia: A systematic review and meta-analysis of randomized controlled trials. Psychiatry Res, 271, 516-525.

-Park, S., Bak, A., Kim, S., Nam, Y., Kim, H. S., Yoo, D.-H., & Moon, M. (2020). Animal-assisted and pet-robot

interventions for ameliorating behavioral and psychological symptoms of dementia: A systematic review and meta-analysis. Biomedicines, 8(6), 150.

-Sheikh, J. I., & Yesavage, J. A. (1986). Geriatric Depression Scale (GDS): recent evidence and development of a shorter version. Clinical Gerontologist: The Journal of Aging and Mental Health.

-UN. (2020). World Population Ageing 2020 Report. Department of Economic and Social Affairs. Retrieved from https://www.un.org/development/desa/pd/sites/www.un.org.development.desa.pd/files/undesa_pd-2020_world_population_ageing_highlights.

-Zelinski, E. M., & Gilewski, M. J. (2004). A 10-item Rasch modeled memory self-efficacy scale. Aging Ment Health, 8(4), 293-306.

Dear Chairman of Daegu University Bioethics Committee

대구대학교
DAEGU UNIVERSITY
